# Evaluating social contract theory in the light of evolutionary social science

**DOI:** 10.1017/ehs.2021.4

**Published:** 2021-01-20

**Authors:** Paul Seabright, Jonathan Stieglitz, Karine Van der Straeten

**Affiliations:** 1 Toulouse School of Economics, University of Toulouse Capitole, Toulouse, France; 2 Institute for Advanced Study in Toulouse, University of Toulouse Capitole, Toulouse, France

**Keywords:** Political philosophy, small-scale societies, social contract, Hobbes, Locke, Rousseau, Darwin

## Abstract

Political philosophers have long drawn explicitly or implicitly on claims about the ways in which human behaviour is shaped by interactions within society. These claims have usually been based on introspection, anecdotes or casual empiricism, but recent empirical research has informed a number of early views about human nature. We focus here on five components of such views: (1) what motivates human beings; (2) what constraints our natural and social environments impose upon us; (3) what kind of society emerges as a result; (4) what constitutes a fulfilling life; and (5) what collective solutions can improve the outcome. We examine social contract theory as developed by some early influential political philosophers (Hobbes, Locke and Rousseau), who viewed the social contract as a device to compare the ‘natural’ state of humans with their behaviour in society. We examine their views in the light of recent cross-cultural empirical research in the evolutionary social sciences. We conclude that social contract theorists severely underestimated human behavioural complexity in societies lacking formal institutions. Had these theorists been more informed about the structure and function of social arrangements in small-scale societies, they might have significantly altered their views about the design and enforcement of social contracts.

## Graphical abstract

**Figure:**
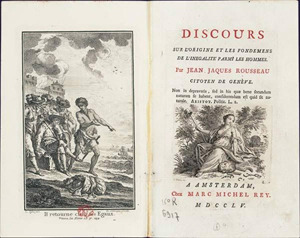

**Social media summary:** Social contract philosophers overlooked the central role of informal social institutions in governing human behavior.

## 1. The implicit empirical underpinnings of political philosophy

### 1.1. Empirical claims about human society

From its earliest beginnings, political and social philosophy has been not only conceptual but also empirical, albeit at a very general level. This is true not only of those usually considered political philosophers (such as Plato, Aristotle, Confucius, Machiavelli, Hobbes, Locke, Voltaire, Montesquieu, Rousseau, Hume and Mill), but also and especially of sociologists, economists, historians and essayists such as Herodotus, Thucydides, Ibn Khaldun, Montaigne, Gibbon, Smith, Malthus, Ricardo, Marshall and Weber, all of whom made contributions that have had a lasting impact on political philosophy, broadly considered.

Such writers as these have expressed their opinions about the facts of at least five main matters, namely:

1. What motivates human beings – what makes us think and behave the way we do?
2. What constraints our natural and social environments impose upon us?
3. What kind of society emerges as a result?
4. What is ‘good’ for human beings, in the sense of constituting a fulfilling life, whether or not we are aware of it?
5. What collective solutions might be found to improve the outcome.

For the purposes of this paper, we will refer to each set of views about these five matters as ‘a theory of human nature’. Although these views contain inescapable elements of value judgement, they are also grounded in broadly empirical claims about what human beings and their societies are like.

While this framing does not necessarily correspond to how political philosophers have expressed themselves, it provides a general conceptual framework that can be empirically evaluated, and it shares core features with frameworks of evolutionary social scientists including Darwin. When Aristotle wrote ‘Man is a political animal’ (*Zoon Politikon*), did he mean that man is motivated by political aims, or that a life of political action is the only worthwhile life for man? Probably he meant both. However, like many other political philosophers, he developed his views on the basis of generalisations from his own and others’ observations.

So did Hobbes, when he claimed that the life of man in the state of nature was ‘solitary, poor, nasty, brutish and short’ (in *Leviathan*, Part 1, Chapter 13), or Rousseau, when he claimed that ‘man is born free, and he is everywhere in chains’ (in the opening sentence of *On the social contract*). Our contention in this paper is that it is time to update many of these generalisations from observation in the light of more recent empirical research, beginning with Darwin (1871). We do not aim to provide a systematic, let alone exhaustive, review; instead we select a few illustrative generalisations that have profoundly influenced contemporary academic and public discourse on political philosophy, and for which sufficient research within the evolutionary social sciences exists to assess their empirical validity.

In writing thus of ‘generalisations from observation’ we do not mean to imply that political philosophers have been innocent empiricists whose only shortcomings are due to their having lived before the publication of the latest high-quality scientific research. Quite the contrary: many political philosophers have been engaged in consciously partisan, even propagandist projects. Some have been tutors or counsellors to political rulers – most famously Aristotle, who was tutor to the future Alexander the Great, and Thomas Hobbes, who was tutor to the future King Charles II (who later helped shield his former tutor from charges of atheism). Many others, including Ibn Khaldun, Niccolo Machiavelli, John Locke and Adam Smith, have enjoyed enough social proximity to those wielding political power for us to be wary of considering their theories to be free of partisan intent. Note that Machiavelli himself was well aware of this fact, and writes: ‘The prejudice which is entertained against the people arises from this, that any man may speak ill of them openly and fearlessly, even when the government is in their hands; whereas princes are always spoken of with a thousand reserves and a constant eye to consequences’ (*Discourses on the first decade of Titus Livius*, Book 1, Chapter 57). Arguably one of the strongest arguments for the existence of universities is to make scholars less directly dependent on the favour of rulers whose propagandists they may be tempted to become.

Even without personal incentives to argue for specific political arrangements, philosophers have been profoundly influenced by the economic and social circumstances in which they lived, to the point where their taking a certain view on human nature or the nature of society may be at least as fruitfully illuminated by these circumstances as by the evidence available to them at the time. That this is now a truism owes a great deal to the influence of historians of political thought such as Quentin Skinner and the Cambridge School (Skinner, 1969). Our purpose here is in no way to downplay these economic or social influences, nor to indulge in pointless counterfactual speculation about what Aristotle might have argued if only he had had advance access to *Nature* or *Evolutionary Human Sciences*.

Nevertheless, among the reasons why the works of such political philosophers have had such persuasive force over the centuries has been that they are considered to embody fundamental views about the nature of human beings and their place in society that appeal to diverse readers. Keynes's remark on the last page of *The general theory* that ‘Practical men who believe themselves to be quite exempt from any intellectual influence, are usually the slaves of some defunct economist’ (Keynes, 1936) can be applied with even greater vigour to defunct political philosophers, who may exert an unacknowledged influence on views about human nature and society that deserve to be openly examined and reviewed.

A particularly well known example is the *tabula rasa* theory of the human mind, which dates back at least to Aristotle's *De Anima*, but is best known today through the writings of John Locke (1689a). The theory has been sufficiently influential that Steven Pinker devoted an entire book to demolishing it (Pinker, 2002) and both Locke and Pinker are widely cited today in the literature on individual and social learning.

It may matter less whether views such as these are accurately attributed to the authors concerned, and still less whether modern readers understand why they were adopted in the first place. Such views can be assessed against later available evidence, even if we accept that the factors that may have contributed to their development and propagation are rarely reducible to empirical conviction alone.

We have a specific reason for evaluating select empirical content relevant to matters 1–5 above. These matters correspond to a way of analysing social processes that has been used fruitfully within a number of social sciences, and which we can call *equilibrium analysis*. It has been most explicitly deployed in economics but is also used in political science, evolutionary anthropology and biology, as well as some parts of quantitative sociology and economic history. Its strength consists in allowing the facts under 1 and 2 to explain the outcomes 3, which can be compared against the standard of evaluation 4. Proposals for improved outcomes 5 then have to be checked for consistency with 1 and 2 and shown to be feasible in spite of the actual tendency of society to produce inferior outcomes 3. Outcomes 3 represent equilibria of the social processes characterised by 1 and 2, and the alternative outcomes 5 have to be possible equilibria as well. The word ‘equilibrium’ is not always used explicitly in such analysis, so it is worth defining explicitly.

An equilibrium is a state of society which, given the motivations and constraints of its members, would tend to persist in the absence of some external disturbance. Note that an equilibrium is not necessarily a desirable outcome under any reasonable criterion: indeed perhaps the most famous description of a political equilibrium is George Orwell's horrifying injunction in his novel *1984* to ‘imagine a boot stamping on a human face, forever’. Yet there is an important reason for focusing on equilibria: if we try to implement political solutions that are not equilibria, they will not last.

### 1.2. The interest of the social contract approach

The range of possible political philosophers we might have chosen to cover is vast. For this paper we draw examples from three thinkers (Hobbes, Locke and Rousseau) who developed a ‘social contract’ approach to political philosophy. Our aim here is certainly not to propose a synthetic summary of their philosophical thought, but simply to give our subjective interpretation of an illustrative subset of their ideas about human nature.

These three writers articulated with particular clarity a distinction between attributes of human motivation and behaviour in the ‘state of nature’ vs. those that were the products of society with formal institutions. By ‘formal institutions’ we refer to those created by humans to govern human behaviour; they comprise written guidelines (e.g. laws, charters, constitutions) that are enforced by authorities. This distinction between humans in the state of nature and in society with formal institutions was radically novel in the history of political thought, but has remained an important part of our thinking about human behaviour ever since.

Whether we look at ancient thinkers such as Aristotle or Confucius, or more modern ones such as Marsilius of Padua or Machiavelli, before the writings of the social contract theorists humans were unimaginable as abstracted from society. For Confucius, for example, humans are inextricably embedded in a network of social relationships, which define who they are as well as their duties and moral obligations. For Machiavelli, ever-changing relations of power, submission and subjugation between individuals are among the defining features of life in society. For both of them, there would be no point in thinking about humans in isolation, independently of the social and political context in which they live.

This means that, in examining the work of these thinkers, we will distinguish under item 3 between the kind of society which emerges when human beings interact in a state of nature, and that which emerges in a modern society with formal institutions. These thinkers drew wildly different conclusions from this contrast; for Hobbes, the state of nature involved such fear and potential for violence that even the institutions of a repressive monarchy were much to be preferred, while for Rousseau the institutions of modern society (and notably those of private property) were responsible for far greater evils than any in a state of nature. Neither author explicitly used the notion of equilibrium that we described above, but for both of them the underlying idea is there. Societies are the outcome of social processes resulting from the interaction of people who have certain characteristics; if we want to work for better outcomes we should forget about trying to change human nature, and concentrate instead on changing the conditions under which people interact.

This should clarify why we focus on empirical evidence from small-scale societies. Compared with industrialised and agricultural societies, small-scale societies – including foragers, horticulturalists, pastoralists and subsistence-level societies with mixed economies (e.g. forager–horticulturalists) – possess more similar social and ecological features that were typical of more than 99% of the evolutionary history of the genus *Homo* (prior to the first agricultural revolution around 12,000 years ago). These features include: subsistence lifestyle characterised by minimal technology; limited energy availability (a high physical activity level relative to consumption); limited material wealth; high fertility; frequent resource pooling within residential settlements; and an absence of formal institutions.

We acknowledge that the term ‘small-scale societies’ is inadequate to capture the range of variation in production technology, social organisation and hierarchy, demography, responses to external social processes and other features characterising both contemporary and past human societies (Reyes-Garcia et al., 2017). Indeed, under certain ecological conditions, foragers are known to have been relatively sedentary, produced food surpluses which were stored, been territorial, owned property and had marked status differentials (Rowley-Conwy, 2001).

Nevertheless, for our present purposes we can say that small-scale societies illustrate one kind of equilibrium in which humans may find themselves, and large-scale societies illustrate another. The contrast between the two may illuminate what difference formal institutions can make. However, whereas evidence from large-scale modern societies is everywhere around us, evidence from small-scale societies has been much less easily available, and thus lends itself more to inaccurate characterisation.

We have two reasons for focusing on small-scale societies, and neither depends on claiming that the thinkers we examine believed that a state of nature had ever really existed. The first reason, which is not really controversial, is that these are the only observable instances available of human societies that function without formal institutions, which is the closest set of real conditions to those which the social contract theorists had in mind. The second, which is much more controversial, is that many of the conditions of small-scale societies have persisted since the origin of modern humans, constituting a physical and social environment against which humans have been evolving under the influence of both natural and cultural selection. To the extent that there are common features of psychology and behaviour in such societies, they may come closest to constituting a ‘human nature’ that can be compared with the human nature to which our various philosophers appealed.

Finally, a word about Darwin. As is well known, Darwin avoided addressing questions about human evolution in *The origin of species*, not because he believed them to be unimportant, but because he was afraid that controversy over the social, political and theological implications of his theory of evolution by natural selection might impede the scientific evaluation of the theory itself. By the time he came to write *The descent of man* he had realised that such controversy was unavoidable and was better addressed directly. In the latter book he made very clear that natural selection had shaped not only human morphology but also human behaviour, and the subsequent century and a half has shown what a powerful insight that was.

For much of the twentieth century, unfortunately, explanations of human behaviour in terms of environmental factors were considered substitutes for rather than complements to explanations drawing on natural selection, and this undoubtedly explains why political philosophy has not made more explicit use of the insights of the Darwinian approach. It is now much easier to accept that humans are flexible learners who are massively influenced by signals from their natural and social environments because natural selection has shaped them to be this way. The work of Robert Boyd and Peter Richerson has been particularly influential in this regard – see Boyd and Richerson (1985) and Richerson and Boyd (2005). In this spirit we hope that an attempt to draw out some implications for political philosophy from empirical research in evolutionary social science, in particular cross-cultural human behavioural variation, is an appropriate tribute to *The descent of man* on the 150th anniversary of its publication.

We structure this paper as follows. In Section 2, we set out the views of the three social contract theorists about the five main matters of political philosophy we outlined above. In Section 3 we summarise the ways in which recent research in evolutionary social science can cast light on these views. Section 4 answers some general questions about how the circumstances of the state of nature should be interpreted. In Section 5 we briefly summarise and conclude.

## 2. The social contract theorists

### 2.1. Common features of the social contract

While there are many differences between the social contract theorists, we begin by focusing on their commonalities. This can best be characterised as an attempt to establish the principles of legitimate political power through a rational and materialistic approach. Importantly for our purpose, these philosophers explicitly base their system on some description of human nature, as emphasised in the concept of a ‘state of nature’. The root of their philosophical system is in a sense anthropological.

The purpose of this exercise is to seek a basis of power that is less debatable than divine law, and less arbitrary than the simple use of force. To do so these theorists turn to the legal concept of contractual agreement based on mutual consent. The idea of the contract is clearly borrowed from law. From the Latin ‘societas’, the word ‘society’ originally refers to a contract whereby individuals pool property and activities, and in which the partners undertake to share any loss or benefit that may arise from this association.

The fundamental shared concepts of social contract theorists include the state of nature, and two types of contracts, those of association and of government:

- The state of nature – the state of humans having no other bond between them than their common quality of being human. Different thinkers hold different views as to what other qualities were associated with this state; although all thought of humans in this state as ‘equal’ in some respects, the character of this equality differed substantially.
- The ‘contract of association’ – the contract of individuals among themselves when they decide to unite to confer on a single person or an assembly the task of making decisions in such a way that these decisions are considered to be the will of all.
- The ‘government contract’ – the voluntary surrender of some individual liberty and the promise to obey the government.

Social contract theories differ according to their conception of the state of nature and their analysis of the two contracts. Importantly, they all share a similar methodology: start from a description of human nature (as perceived to be real, or abstractly in the state of nature), and derive from these foundations, in a deductive way, the principles of legitimate political power. All were inspired by natural sciences and mathematics (in particular Hobbes by geometry and Rousseau by chemistry).

In addition to a conscious distancing from scholasticism and from religious justifications for political legitimacy, social contract theories also broke with ancient philosophy and in particular with Aristotle's *Politics*, which established social organisation on moral principles and on natural rights that existed prior to formal societal institutions. For these social contract theorists it is not true that ‘man is by nature a political animal’. Man becomes social only by his own will. The city-state does not exist by nature, but results from a convention.

We now look at ways in which the details of the social contract theories varied between thinkers.

### 2.2. Hobbes

Instead of starting from a (claimed) revealed truth, Hobbes bases his social contract theory on his conception of human nature, in one of the first attempts at the secularisation of political thought. In *Leviathan* (published in 1651), Hobbes advances several claims:

- man in the state of nature is fundamentally fearful and driven by passions;
- individuals are fundamentally equal;
- for fear of violence, they will willingly abdicate their right of nature in favour of an absolute sovereign who will guarantee public peace using the power of repression at his disposal.

In terms of the five matters we outline above, the Hobbesian view of human nature is that:

1. Humans in the state of nature seek only to ensure their own preservation, by all means, and are driven by passion, particularly for power (‘in the first place, I put for a general inclination of all mankind a perpetual and restless desire of power after power, that ceases only in death’; chap. XI). Moreover, men are essentially equal in strength and capacity (‘Nature hath made men so equal in the faculties of body and mind as that, though there be found one man sometimes manifestly stronger in body or of quicker mind than another, yet when all is reckoned together the difference between man and man is not so considerable as that one man can thereupon claim to himself any benefit to which another may not pretend as well as he. For as to the strength of body, the weakest has strength enough to kill the strongest, either by secret machination or by confederacy with others that are in the same danger with himself’; chap. XIII).
2. The main constraint on individuals is the capacity of others to inflict violence on them. In contrast, they are not constrained in their ability to inflict violence on others. Hobbes considers that natural law (*jus naturale*) gives the individual absolute freedom to do whatever his power allows him to do: ‘Every man has a right to everything’. Natural law depends on the strengths of each individual. Any idea of justice will only appear with formal laws: there is no justice to the state of nature (contrary to what Locke argues, for example).
3. The result is a situation of chaos with rife potential for civil war. Permanent insecurity and danger characterise this natural state, which Hobbes sees as a state approximating that of war. No one is ever really at peace in this situation, and in order to defend oneself, it is necessary to attack others. The state of nature is therefore characterised by struggles ‘of each against each’.
4. In these circumstances, the overwhelming need of human beings is security.
5. The means to achieve security can only come in the form of law – not ‘natural law’, which is a source of freedom rather than constraint, but social law. Consequently, it is inevitable that each individual decides to enter into a contract with each of the others, in order to abdicate part of their power in favour of a common authority with absolute power, the State, or Leviathan, described as a ‘mortal god’. Only a strong authority is capable of guaranteeing everyone the preservation of their lives and property. Put another way, government, according to Hobbes, must flow from a covenant of each person to each other – not to the sovereign – in which all cede to the sovereign their right to govern themselves and their freedom so that the will of the sovereign brings the wills of all individuals to one single will. It is important to note that the contract is at this stage ‘horizontal’, not ‘vertical’ (the contract of association). ‘The only way to erect such a common power, as may be able to defend them from the invasion of foreigners, and the injuries of one another, and thereby to secure them in such sort as that by their own industry and by the fruits of the Earth they may nourish themselves and live contentedly, is to confer all their power and strength upon one man, or upon one assembly of men, that may reduce all their wills, by plurality of voices, unto one will’ (chap. XVII).

Hobbes explains that the state can take three forms, depending on how power is exercised: by a single man in a monarchy, by the assembly of citizens in a democracy, or by a small circle in an aristocracy (chap. XIX). He is a strong advocate of absolute monarchy, considering this type of regime to be the most suitable for ensuring peace and security for the people (II, 19). The two characteristics of the government contract according to Hobbes are: the fact that submission must be total; and the fact that the ruler himself is not bound by the contract (i.e. his power is absolute). Total submission on the one hand and absolute power on the other are the *sine qua non* conditions of a civil state – that is, a state of peace. Indeed, the mere possibility of recourse against the sovereign would lead to a return to the struggle of each against each.

### 2.3. Locke

Locke shares with Hobbes his contractual doctrine of the state, and also his determination to found this doctrine in opposition to a previously dominant theory of the divine right of kings revealed by scripture. In the *First treatise* he spends most of his time demolishing the *Patriarcha* of Sir Robert Filmer, which was one of the most influential texts defending such a doctrine of scriptural revelation. Nevertheless, Locke develops the argument in a very different way from Hobbes. Starting from very different premises about the state of nature, he reaches very different political principles (*Two treatises of government*, Locke, 1689b). This reminds us that the content of these philosophers’ claims about the state of nature plays a crucial role in justifying their normative conclusions.

For Locke, the state of nature is a state of harmony and reasonable freedom. He describes it in Chapter 2 of the Second Treatise as:

a state of perfect freedom to order their actions, and dispose of their possessions and persons as they think fit, within the bounds of the law of Nature, without asking leave or depending upon the will of any other man. A state also of equality, wherein all the power and jurisdiction is reciprocal, no one having more than another, there being nothing more evident than that creatures of the same species and rank, promiscuously born to all the same advantages of Nature, and the use of the same faculties, should also be equal one amongst another, without subordination or subjection, unless the lord and master of them all should, by any manifest declaration of his will, set one above another, and confer on him, by an evident and clear appointment, an undoubted right to dominion and sovereignty.

Individuals in the state of nature enjoy two powers and a fundamental right: the power to ensure their own conservation; the power to punish anyone who threatens their life; and the fundamental right of ownership limited to what is necessary for its conservation. Contrary to Hobbes, Locke recognises natural rights: he argues that people have rights, such as the right to life, liberty and property, that have a foundation independent of the laws of any particular society. This is not, of course, an empirical disagreement with Hobbes in the ordinary sense of the term, but rather a difference of values. Nevertheless, Locke's view of the state of nature as harmonious (an empirical claim) helps to make the claim that people in it enjoy certain rights less contrived than it would be in a state of total fear such as that described by Hobbes.

The right to property contains two interesting elements. First there is a version of a labour theory of value:

Whatsoever, then, he removes out of the state that Nature hath provided and left it in, he hath mixed his labour with it, and joined to it something that is his own, and thereby makes it his property (Second Treatise, Chapter V, paragraph 27).

The second is a requirement, which acts as a qualification to the labour theory of value, that the act of appropriation should not harm the opportunities for appropriation by others:

For this ‘labour’ being the unquestionable property of the labourer, no man but he can have a right to what that is once joined to, at least where there is enough, and as good left in common for others (Second Treatise, Chapter V, paragraph 26).

In terms of the five matters we outline above, Locke's view of human nature is as follows:

1. Locke's view of the fundamental motivation of humans is different from that of Hobbes in that the passion for power is less dominant. People are more bourgeois and less martial than for Hobbes (Macpherson, 1962) – violence is more the product of the wish to steal others’ resources than to exercise domination over them (nevertheless, for a reminder of Locke's awareness of the recent history of political and especially religious violence, see Tully (1980, 1993)).
2. Locke also differs from Hobbes in that he considers natural law to constrain human behaviour in the state of nature. Although this represents a difference of values and not of simple fact, it is a claim that makes more sense in a relatively peaceful state of nature than it would in a Hobbesian struggle of all against all.
3. Although the state of nature is indeed more harmonious than it is for Hobbes, it is still regularly threatened by insecurity. It also contains enough material abundance that the right to appropriate nature by mixing it with one's labour does not regularly prevent their being ‘enough and as good left in common for others’.
4. Security is necessary to achieve peace and prosperity, which are fundamental goods for Locke (he places more value than Hobbes on prosperity).
5. Individuals therefore enter into civil status by a contract of association (mutual consent) and a contract of conditional submission. Unlike for Hobbes this submission is only conditional. Locke believes that no legitimate (i.e. freely consented) government can be an absolute government. No one would be foolish enough to consent to give up all their rights, because then the state of society would be worse than the state of nature (the same idea will be found in Rousseau). Locke's idea is that, in civil states, the rule is that of the majority and not of an all-powerful authority. Furthermore, the government contract is dissolved as soon as the majority considers the government to be inadequate, i.e. unable to provide security.

### 2.4. Rousseau

In *The social contract* (published in 1762), Rousseau's stated objective is also to determine the source and principles of legitimate political authority. In the first lines of Book 1, he states his aim very clearly: ‘My purpose is to consider if, in political society, there can be any legitimate and sure principle of government, taking men as they are and laws as they might be’ (Rousseau, 1762).

The precise character of the state of nature is not much discussed in that book, but it had been treated extensively in his earlier *Discourse on the origin and basis of inequality among men*, published in 1755, five years before *On the social contract*. The entire first part of this earlier text is devoted to the state of nature, which is described as ‘simple, unchanging and solitary’ (Rousseau, 1755). Individuals do not think, do not communicate beyond the most rudimentary means (gestures) and live essentially in the present. The only feelings in this state of nature are a concern for self-preservation (‘amour de soi’), as well as a minimal sense of pity for others. The cohabitation of the two guaranteeing a certain balance, none wanting to inflict unnecessary harm on others.

For Rousseau, this state of nature disappeared long before his own time. However, Rousseau looks for examples of indigenous peoples to discover what could give us the best idea of this state. The closest example of this state of nature is according to him that of the ‘Caribbean savages’, about whom his information was of course somewhat limited.

Rousseau believed that, in the state of nature, individuals live in the present, and find all of the resources to meet their basic needs. Individuals are essentially solitary, the family itself being for Rousseau only a notion invented later.

The state of nature is a state where inequalities are minimal. It is society and the associated formal institutions that create and exacerbate inequalities, first by creating new inequalities, the first and most important of all being through the invention of property, but also by exacerbating natural inequalities of talent, for example through formal schooling. Moreover, in the state of nature, individuals have very little power over each other; indeed, even inequalities of strength are not enough to subjugate each other, because in this state of nature, anyone can always leave.

The second part of the *Discourse* is devoted to telling the story of this exponential progression of inequalities, which begins, as noted above, with the invention of property. Rousseau traces its origins and later development back to the inventions of agriculture and then metallurgy. The appearance of property, the increasing inter-dependencies which resulted from the division of labour, and the feeding of passions born of other men's trade, made conflicts between men more and more permanent and violent. Then came the creation of magistrates, exercising political superiority over others, until the appearance of the ultimate form of inequality: despotism and absolute submission.

At this point Rousseau attacks both Hobbes and Locke head-on. He blames both for confusing various states of civil society with the state of nature. Hobbes takes as a state of nature the struggles of all against all to justify absolutism. In addition, Locke integrates the notion of property into the state of nature, which leads him to defend the social contract as the means of protecting this property. For Rousseau, on the contrary, it is this very notion of property that marks the exit from the state of nature.

In terms of the five matters we outline above, Rousseau's view of human nature is as follows. Rousseau gives his first definition of human nature in Chapter II of Book 1:

This common liberty is a consequence of man's nature. Man's first law is to watch over his own preservation; and as soon as he reaches the age of reason, he becomes the only judge of the best means to preserve himself; he becomes his own master.

The first and main motivation of individuals is self-preservation; and as each can best judge what is best for them, liberty is an absolutely essential feature. This feature is common with Hobbes and Locke. Later, Rousseau diverges from these authors, as he describes the state of nature as a state where individuals live in isolation, ‘entirely complete and solitary’ (Chapter VII, Book 2).

Rousseau also had ideas about the extent of human preferences for the present over the future. In the *Discourse* he wrote that the soul of

the savage man … which nothing disturbs, dwells only in the sensation of its present existence, without any idea of the future, however close that might be, and his projects, as limited as his horizons, hardly extend to the end of the day. Such is, even today, the extent of the foresight of a Caribbean Indian: he sells his cotton bed in the morning, and in the evening comes weeping to buy it back, having failed to foresee that he would need it for the next night.

Quite where Rousseau obtained this implausible information he did not say.

Rousseau is evasive about the reasons underlying the transition from the state of nature, where individuals lived in isolation, to living together. His objective is not to describe how human beings ended up living in large groups, but what the legitimate ways are of funding and organising such societies.

The answer to this question is given in the first sentence of Chapter 1: ‘Man is born free; and he is everywhere in chains’. This is the main topic of the *Discourse on the origin and basis of inequality among men*, describing the modern condition as dominated by inequality, dependency, war and misery. Almost everywhere, humans have lost their autonomy and freedom.

For Rousseau, entering into a harmonious social community has the potential ‘to change human nature, to transform each individual, who by himself is entirely complete and solitary, into a part of a much greater whole’ (Chapter 7 of Book II). This answer is essentially the same as that given by Hobbes and Locke.

Given that autonomy and liberty are essential qualities of an individual, the only legitimate foundation of society is the unanimous consent of all: ‘the Social Contract’. The whole population taken together forms the sovereign; it represents the general will and possesses the legislative power. The government, distinct from the sovereign, deals with the application of law and other particular matters. Rousseau forms the theory of the ideal constitution. Yet he remains always wary that this is an unstable state. Because of the first law of human nature, self-interest will be present, and creates at least two kinds of tensions: the first between each individual and the sovereign (the general will), and the second between the sovereign and the government, the latter always being tempted to act in its own interest.

## 3. Empirical evidence

### 3.1. What motivates human beings

Under what conditions is human social behaviour motivated by selfishness, cooperation, altruism and spite? The answers are central for evaluating the validity of descriptions of individuals in the state of nature, and reasons underlying demands for social contracts. Whether from an evolutionary economic perspective selfishness and cooperation (both of which entail fitness benefits for actors) are easier to explain than altruism and spite (which entail fitness costs for actors) has been the topic of much debate since roughly the middle of the twentieth century (see Alger & Weibull, 2019, for a review of theoretical models of preference formation in social interactions).

Modern research in behavioural economics has greatly enhanced our ability to adjudicate rival causal claims about the relative importance of selfishness and altruism, in particular in individual human motivation. Mainstream economics assumed that individuals were selfish unless given incentives to behave otherwise. The long-standing claims by other disciplines including anthropology, biology and psychology for the existence of what are now called ‘social preferences’ (not only positive ones such as reciprocity but also negative ones such as envy) were dismissed by traditional economists as just the social manifestation of rational self-interest.

For example, it was commonly argued that people might behave apparently altruistically just because of the selfish benefits to be gained from others’ response to this behaviour. The result was that different branches of the evolutionary social sciences made different assumptions about human motivation as a matter of methodological preference or other considerations (such as mathematical tractability), and different flavours of political philosophy made different assumptions about human motivation out of equally unchallenged prior conviction. All sides appealed abundantly to anecdotes, and few efforts were made to look to systematic empirical enquiry to settle their differences.

This changed, in part, with laboratory experiments in the field of what has now come to be called behavioural economics. What these experiments made possible was to observe how individuals behaved when interacting with anonymous others (e.g. owing to double-blinded study designs), and whom therefore they would never knowingly encounter again – thereby ruling out strategic motivations for displaying apparently altruistic or cooperative behaviour. Yet instead of revealing that individuals in such contexts behave purely self-interestedly, the experimental literature has shown that under a wide range of circumstances they:

- make altruistic donations to individuals they will never meet (Forsythe et al., 1994);
- respond to the behaviour of others with strong reciprocity – being generous to those who have been generous to them, and mean to those who have been mean to them (Fehr et al., 1993, 1997; Fehr & Schmidt, 1999);
- are willing to contribute to public goods, but quickly reduce their willingness if they believe it is being exploited by others who are free riding (Fehr & Gächter, 2000);
- are willing to punish those whom they believe to be cheating or unjust to others, even at a cost to themselves and even if they themselves have not been directly harmed by the cheating or unjust act (Fischbacher, 2001; Fehr & Fischbacher, 2004).

It is important to emphasise that there is a wide variety of findings, and this literature has not replaced the uniform view that human motivation is selfish by a similarly uniform view that it is characterised by any particular configuration of social preferences. Nevertheless, the validity of the rational egoism view is now seriously in doubt. Legitimate questions can be and have been posed about the ecological validity of such anonymous experiments (Pisor et al., 2020), the interpretation of findings from ecologically valid studies (such as natural field experiments; see Winking & Mizer, 2013 for an example) and their relevance to small-scale societies. Indeed, the vast majority of economic experiments have been conducted with subjects from so-called WEIRD populations (Western, Educated, Industrialised, Rich and Democratic – see Henrich, 2020). Precisely because of such concerns, major efforts have been made in recent years to conduct economic experiments in a range of small-scale and other non-WEIRD societies, and results indicate substantial heterogeneity (Engel, 2011).

Some of the behaviours highlighted above (e.g. willingness to punish others at a cost to oneself) have been found in populations from many different societies and classes within societies (Henrich et al., 2001, 2006). There is substantial variation among individuals in any society.

All known study populations contain some purely self interested individuals as well as others with a variety of types and degrees of social preferences including altruism and spite. Yet nobody can any longer maintain that the hypothesis of rational self-interest is descriptively accurate for humans, although there remains much room for argument about how useful this hypothesis can be as a working approximation in certain political and economic contexts.

It is a somewhat subtle matter to judge just how much of a revelation these findings would have seemed to the philosophers whose views we have outlined. Locke and Rousseau came closest to thinking that man in the state of nature corresponds to the picture of *Homo economicus* that modern evolutionary social science has shown to be so incomplete; to the extent that more other-regarding motivations were possible these would have been considered to be the result of social pressures. Ancient thinkers such as Aristotle or Confucius would doubtless have been much less surprised, since they explicitly accepted that humans could be motivated by other than selfish objectives. Hobbes, in contrast, already admitted other forms of motivation, but chiefly the more destructive kinds such as spite. He would probably have been surprised to discover that humans are regularly capable of behaving altruistically to strangers they never expect to meet again.

With respect to time-preference, there is some evidence, drawn from studies of material preferences using forced-choice paradigms, that individuals in forager–horticultural societies are more present-oriented and discount future rewards (Kirby et al., 2002); this may deter material wealth accrual. However, it is hard to know whether this reflects intrinsic time-preference or different sets of beliefs about the probability of receiving future rewards. And there is certainly no evidence at all suggesting that inhabitants of small-scale societies are unable, as Rousseau thought, to foresee their material needs 24 hours in advance.

### 3.2. What constraints we face from our natural and social environments

The social contract theorists we have considered believed that the natural state of individuals was anything but social. Hobbes famously wrote that the state of man was ‘solitary, poor, nasty, brutish and short’. Rousseau also considered the state of nature to be solitary.

This picture is radically at odds with what we now know to be the shared characteristics of all known human societies. Across small-scale societies there is a modal pattern of social organisation characterised by a three-generational system of resource flows (including co-resident offspring, par-ents and grandparents), a sexual and age-graded division of labour within long-term adult pair bonds (Alger et al., 2020) and high levels of cooperation between kin and non-kin (Kaplan et al., 2000). Human hunter–gatherers were probably highly inter-dependent long before the invention of agriculture, contrary to Rousseau's claim that agriculture and its associated divisions of labour paved the way for high inter-dependencies.

Despite the popular idea that hunter–gatherers are organised in a system of small groups of coresiding kin, their social structure is actually quite fluid and comprises networks of interaction between spatially and genetically distant individuals that extend far beyond a local residential group (Bird et al., 2019; Hill et al., 2011; Migliano et al., 2017; Wiessner, 1982).

In the archaeological record, ‘base camps’ – which reflect key organisational components of forager sociality facilitating cooperation – are evident at least 400,000 years ago (Kuhn & Stiner, 2019).

Similarly, genetic evidence of interbreeding between early modern and archaic hominins reveals the formation of expansive kinship ties, and possibly cooperation, at least 100,000 years ago (Kuhlwilm et al., 2016).

Modern psychological and behavioural evidence supports this impression. Experimental studies suggest that we have an evolved cognitive specialisation for reasoning about social exchange and for social learning (e.g. Cosmides & Tooby, 1992; Derex & Boyd, 2018; Derex et al., 2019; Sugiyama et al., 2002), which is not expected for a ‘solitary’ organism. Likewise, analysis of systematic behavioral observation data from a large sample of Tsimane forager–horticulturalists of Bolivia (*n* = 50,349 instantaneous scans of 608 individuals) shows that across all sex and age categories, only 2.4 daylight hours per day are spent ‘alone’, defined here as either not within 3 metres of at least one other individual, or not involved in a conversation (unpublished data).

Not all of this interaction has a positive connotation, of course – far from it. Despite Rousseau's insistence that humans outside of ‘civilised’ society as he knew it were: ‘wandering in the forests, without war … equally without any need of his fellow men and without any desire to hurt them’ (Rousseau, 1755 [1984]), human warfare probably pre-dated agriculture, sedentary residential arrangements and the formation of larger-scale societies (see Glowacki et al., 2020 and references therein). Whether warfare is indeed more prevalent in non-state vs. state societies is vigourously debated. Interstate warfare has become less common over time in many world regions (Pinker, 2011), although some regions remain chronically entrenched in war (Morris, 2014). The fact that some hunter–gatherer groups traded and intermarried with other groups (Fry, 2005) indicates that inter-group relations among non-state societies are not necessarily hostile (as is the case for our close chimpanzee relatives), but instead could alternate between collaboration and hostility in response to changing incentives. However, Peterson and Wrangham (1997) emphasise the strategic character of much violence even among chimpanzees: the probability that encounters turn violent is highly influenced by perceived fitness costs and benefits of aggression.

### 3.3. What kind of society emerges in communities lacking formal institutions

The word ‘poor’ in Hobbes's description of life in the state of nature does indeed correspond to some aspects of reality in small-scale societies. Perhaps the most important consideration, though, is not average levels of wealth but the equality or inequality in the distribution of that wealth. Because, in general, resources are much more equally distributed in small-scale compared with larger-scale societies, material poverty in small-scale societies may be experienced very differently from poverty in societies with significant storable and heritable wealth and wealth inequalities (Jaeggi et al., 2020).

Wealth can be defined in various ways: (1) material, (2) relational and (3) embodied (Borgerhoff Mulder et al., 2009). The relative paucity of owned, defensible and transmittable material wealth among many hunter–gatherers (exceptions notwithstanding, e.g. Ames, 2003; Rowley-Conwy, 2001) entails comparatively minimal wealth inequality. Relative egalitarianism in material resource access limits the formation of rigid, pronounced gradients in health and longevity along status lines that reliably emerge among humans and other primates (e.g. Kondo et al., 2009; Marmot et al., 1991; Sapolsky, 2005). Minimal wealth inequality diminishes subjective experience of deprivation and subordination and associated adverse health consequences, including chronic psychosocial stress and depression (see Snyder-Mackler et al., 2020 for an overview). Foragers who have been able to intensify resource exploitation and produce storable food surpluses which are then defended and transmitted are exceptions that prove the rule: alongside stable material wealth differentials we observe among some foragers status-graded variation in well-being, including the existence of slavery.

Status hierarchies do indeed exist across diverse small-scale societies, but rather than resulting simply from variation in material wealth, they are often linked to relational wealth (i.e. social ties in marriage, food-sharing and other cooperative networks) and embodied wealth (i.e. physical and cognitive abilities, such as strength and knowledge/skill, underlying variation in food production and reproductive success).

At the same time, as Hobbes intimated, inhabitants of small-scale societies often face undesirable physical conditions including harsh and unpredictable environments (e.g. extreme temperatures, omnipresent insects), predation, diverse infectious diseases (a major cause of mortality), and food and water shortages.

Nevertheless, Hobbes's erroneous characterisation of life in small-scale societies overlooks numerous enjoyable leisure activities within such societies, including storytelling (Schniter et al., 2018; Smith et al., 2017), music-making, singing and dancing (Mehr et al., 2019), sport (Trumble et al., 2012) and communal beer-drinking (Hooper et al., 2013), that have long been posited to play central roles in socialisation, information exchange and/or entertainment, but whose form and function are only recently being understood empirically. Beer drinking is of course a product of post-agricultural societies, but consumption of psychoactive plant substances – which may have been used in various collective activities (e.g. ceremonies, recreation, labour) – may have a longer history that predates the Neolithic (Hagen & Tushingham, 2019).

Notwithstanding the recurring potential for violence in small-scale societies, Hobbes's use of the word ‘brutish’ also seems quite inaccurate to describe the subtle mechanisms deployed in many such societies to manage conflict. Precisely because overt conflict or its threat was frequent, we would expect strong selective pressure to evolve strategies for managing conflict. This point has been explored in particular detail in de Waal (2017).

Boehm (1999) documents the many ways in which the relatively egalitarian social structure of some hunter–gatherers both requires and facilitates respect for individual autonomy. Potentially powerful individuals cannot easily dominate, and if they try they face countervailing pressure from coalitions of others (see Gavrilets, 2012, for a theoretical application). Violence is often shunned in daily life (e.g. Tacey & Riboli, 2014); its sanctioned use is generally reserved for extreme cases (e.g. punishing murderers). Excessive use of violence for punishing norm violators often entails moral outrage (Mathew, 2017) and reputational and other costs to overly aggressive norm enforcers. Wrangham (2019) provides an overview of the strategic use of aggression to discipline individuals who are excessively prone to exercise what he calls ‘reactive aggression’ (i.e. a response to a threat or frustrating event, with the goal being only to remove the provoking stimulus), and suggests that this human tendency played a major role in our physical and psychological evolution.

Among Aka hunter–gatherers of the Central African Republic, individuals ‘cite physical or verbal fighting as one of the worst things one individual can do to another, along with not sharing, stealing food or husbands/wives, and sorcery’ (Hess et al., 2010: 338–339).

In many small-scale societies a common response to conflict is for one or multiple involved parties to disperse to another residential group (for short or longer durations, as Rousseau intimated). Relocation costs are relatively low in the absence of formal property rights. When norms are violated within the group, punishment commonly takes the form of criticism, shaming, ridicule, ostracism, mocking or even joking rather than violence (Wiessner, 2005).

Third-party mediation is another common strategy for peacefully resolving disputes, and conflict resolution is a common function of leaders in small-scale societies (see Garfield et al., 2020 and references therein). Other tactics for minimising aggression include arranging marriages for young girls (e.g. Shostak, 1981), which can serve to reduce male competition and unite in-laws. Maintaining group cohesion and reducing tensions can also be accomplished through trance healing dances and ‘fireside chats’ (Wiessner, 2014).

Even families involved in lethal conflicts can avoid the temptation of enacting revenge, e.g. by portraying the incident as a random and isolated incident (e.g. owing to intoxication), or by regarding an aggressor as mentally unstable and not deserving of further attention.

Finally, Hobbes's characterisation of life in the state of nature as ‘short’ was in important respects mistaken. It is true that recorded human life expectancy has increased linearly by three months per year over the past 160 years (Oeppen & Vaupel, 2002), with improvements in sanitation, nutrition and public health accounting for much of this change. Life expectancy at birth is projected to continue increasing in industrialised countries worldwide through 2030, largely owing to enhanced longevity at older ages (Kontis et al., 2017). By 2030 female life expectancy may exceed 90 years.

Such high survival rates have probably never occurred before in human history. Nevertheless, despite their lower life expectancies, hunter–gatherer and horticultural populations with limited access to medical care and sanitation are likely to reach middle age and older adulthood if they survive early childhood (Gurven & Kaplan, 2007). High infant and child mortality yields a life expectancy at birth of 21–37 years for hunter–gatherer populations, but conditional on survival to age 15 years, the modal age of death for hunter–gatherers, horticulturalists and even eighteenth-century Europeans ranges from 68 to 78 years. Human longevity is therefore not simply an artefact of improved living conditions.

Moreover, many chronic causes of morbidity and prevalent causes of mortality in industrialised populations (e.g. cardiovascular disease, diabetes, obesity, hypertension, also known as ‘diseases of civilisation’) are rare or absent in small-scale societies (e.g. Gurven et al., 2012; Kaplan et al., 2017).

This apparent paucity of non-communicable disease is not a result of short lifespan. Rather, various features of lifestyle such as lean and high-fibre diets free of processed foods, high physical activity levels, minimal smoking and other behaviours are protective factors common to many small-scale societies.

### 3.4. What constitutes a fulfilling life?

It might seem as though empirical evidence would hardly be relevant to the question of the good life for humans, which is essentially a matter of value judgements. However, this ignores the fact that evidence from small-scale societies has yielded some valuable insights into aspects of daily life that are reliably associated with mental health, or with various forms of psychological distress including depression. A philosophical theory of the good life for humans is not just a set of value judgements, but also a (loosely) empirical set of hypotheses about the kinds of activities that lead to humans’ judging their lives to be worthwhile, and that lead to human flourishing. Of course, some philosophers have accorded greater weight than others to the judgements of individuals, who are not necessarily considered the best judges of what is good for them. Some (like Nietzsche) have even attacked the idea that contentment with one's life is a desirable state, considering that various forms of striving are far more noble ideals even if these bring stress and disappointment in their wake.

Nevertheless, it is worth noting that evidence has accumulated in recent years in favour of the view that, although there is much between- and within-societal variation in what individuals consider to be worthwhile forms of living, the majority of individuals derive important benefits from intrinsically social aspects of their lives – their networks of family, friends, colleagues and neighbours. Even if some material circumstances are capable of causing great unhappiness – physical illness is a frequent cause of depression, for example – above a certain level of material comfort the contribution of material prosperity to human fulfilment is relatively unimportant.

Psychologists have recognised that human interdependence shapes the self-concept (Markus & Kitayama, 1991) such that social identity is an essential component of self-concept (‘identify fusion’). Ingroup fusion indeed predicts costly self-sacrifice in economic experiments among subsistence and market-integrated populations (Purzycki & Lang, 2019).

Given the importance in terms of biological fitness of inter-individual transfers of resources and assistance in every phase of the human life course (Lee, 2014), it is likely that human psychological well-being responds to the nature and quantity of those transfers (Stieglitz et al., 2014). In particular, deviations of resource transfers from expectations can affect psychological well-being. Resource flows can be disrupted for various reasons; one principal source of disruptions is the inability to provide support for others owing to disability, illness, or some other permanent or temporary shock. Given that downward resource transfers from older to younger individuals are expected in small-scale societies and that illness and disability become increasingly prevalent with age, the inability to provide and share expected resources can be a principal driver of reduced psychological well-being among aging adults. Limited evidence from small-scale societies (Stieglitz et al., 2014, 2015) suggests that risk of depression increases with age, as health, functional ability and productivity decline, and is not characterised by a ‘mid-life crisis’ as in modern societies (Blanchflower & Oswald, 2008).

### 3.5. Collective solutions

Space constraints prevent us in this paper from reviewing the massive literature on political arrangements in small-scale societies, as well as from distinguishing as much as we would have liked between more or less segmented forager societies (see Garfield et al., 2019, for a substantial review of one key dimension of political arrangements, namely political leadership). However, we can make some observations about the extent to which particular informal social institutions have proven robust in the face of individual incentives to disrupt them.

The work of Boehm (1999) cited above suggested that the relatively egalitarian distribution of both material resources and power in certain small-scale societies was the product not of an absence of competitive instincts among their inhabitants but rather an equilibrium in which those competitive instincts were kept in check by the countervailing power of others. Furthermore, a talent for mobilising such countervailing power was suggested by Boehm to be one of the major adaptive innovations of the human social order under certain conditions. To the extent that that countervailing power was successful, its mobilisation could be considered a significant public good. At least in egalitarian hunter–gatherers, in contrast to the views of Machiavelli and Hobbes, influence is exercised largely through prestige rather than dominance, a distinction emphasised by Henrich and Gil-White (2001).

Small-scale societies have also shown considerable ingenuity in mobilising their members to provide other kinds of public goods, including participation in hunting and defence operations (Boyd, 2017). Such participation is clearly strategic and responsive to the fitness benefits of collective action. In particular, as emphasised by Glowacki et al. (2020), ‘warfare is a strategy by which coalitions of males cooperate to acquire and defend resources necessary for reproduction. This strategy is not the result of a single “instinct” for war, but is instead an emergent property resulting from evolved psychological mechanisms (such as xenophobia and parochial altruism). These mechanisms are sensitive to ecological and social conditions, such that the prevalence and patterns of warfare vary according to subsistence strategies, military technology, cultural institutions, and political and economic relations’.

It is notable that the small-scale societies that have implemented collective action in this manner have all done so in spite of the absence of formal legal institutions, which suggests that the social contract theorists considerably underestimated the ability of human societies to find informal solutions to the problems generated by the state of nature.

Among the chief mechanisms for achieving collective action has been the establishment and enforcement of norms (Boyd, 2017) for policing perceived anti-social behaviour. In small-scale and other societies, theft is perceived as immoral, worthy of firm punishment and damaging to one's reputation (Barrett et al., 2016). This would of course have been less surprising to earlier thinkers – Confucius, for example, laid particular emphasis on righteousness, namely on maintaining integrity in the face of temptation. Relatedly, language in small-scale societies reveals a broadly similar set of normative concerns. A lexical study of ‘human attribute concepts’ (i.e. traits ascribable to humans) in 12 isolated languages spanning most habitable world regions outside of Europe found that jealousy and crookedness are relatively ubiquitous human traits (Saucier et al., 2014). Similarly, honesty and dishonesty are among the most salient cross-cultural indicators of good and bad people, respectively, based on free-list responses capturing local attitudes (Purzycki et al., 2018). Marital infidelity is regarded harshly cross-culturally (Scelza et al., 2020), and is the most commonly cited reason for divorce (Betzig, 1989). Given the importance of inter-dependence for survival throughout human history, it has been hypothesised that the emotion of shame evolved to avoid or minimise social costs incurred from committing immoral acts (Sznycer et al., 2018).

Along with such norms there is widespread acceptance of the idea that virtuous people should be sought out as social partners, and that anti-social individuals should be shunned. ‘Lab in the field’ economic experiments among Hadza hunter–gatherers of Tanzania and Tsimane forager–horticulturalists of Bolivia indicate that, despite substantial residential mobility, cooperators are preferentially connected to other cooperators in social networks (Smith et al., 2018; Stieglitz et al., 2017). In Bwa Mawego, Dominica, men with better altruistic reputations form more same-sex reciprocal labour partnerships than men with poorer reputations (Macfarlan et al., 2012). Finally, the Hadza appear to agree on which traits constitute moral character (i.e. being a hard worker, generosity and honesty) but disagree on which specific camp mates actually exhibit these traits (Smith & Apicella, 2019), suggesting plasticity in individual dispositions depending on context.

In addition to providing public goods and policing anti-social behaviour, individuals in small-scale societies also engage in other kinds of collective activity of a kind that can be described as rituals, the difference being that in rituals the activity is itself constitutive of the collective benefit being provided, rather than merely being instrumental in its provision as is the case for public goods. Although the social contract theorists did not attach much if any importance to ritual, thinkers as diverse as Confucius, Ibn Khaldun (1377) and Durkheim (1915) have argued that rituals promote group cohesion in various ways, including ensuring commitment to collective goals, acquiescence to group traditions (e.g. moral codes, social obligations, institutions) and deference to authority figures. More recent empirical research confirms this. In the Tyvan Republic of southern Siberia, ethnic Tyvans who regularly engage in cairn rites (e.g. making offerings to local spirits by burning incense and leaving money, food or tobacco) are perceived as more trustworthy than ethnic Tyvans, Christian Tyvans and Christian Russians who do not perform such rites, suggesting that ritual increases bonds between practitioners (Purzycki & Arakchaa, 2013).

In Mauritius, participation in painful rituals (e.g. dragging heavy objects attached by hooks to the skin for hours) is associated with greater anonymous charitable donations and more inclusive social group identification (Xygalatas et al., 2013). In southern India, those who partake in collective ritual (e.g. monthly temple worship) are more likely to report between them supportive relationships (e.g. receiving advice or loans) compared with those who refrain from ritual; importantly, ritual participants are also able to maintain supportive relationships with non-participants (Power, 2018). Ritual participants are also perceived as more devout, generous, better advisors and having good character compared with non-participants (Power, 2017a), and are more likely to act in ways that benefit others (Power, 2017b). Together, these recent empirical case studies tend to support Confucius’ proposal that ritual enhances social cohesion, in part by curbing anti-social behaviour and promoting cooperation.

Overall, we can consider that the collective solutions to the challenges that humans encounter fall into two broad categories. The first category consists of those in which the societies informally mobilise the efforts of their members to provide various public goods, including in the form of coordinated defence against or attack of external competitors, or internal dispute management. The second consists of various collective activities whose nature is performative: the action is itself partly constitutive of the benefit and is not merely instrumentally useful. Of course, some kinds of activity (including the collective exercise of violence) may come to acquire a performative character through its repetition over time.

## 4. Some general questions about the social contract approach

### 4.1. Did social contract theorists believe the state of nature had ever really existed?

There is a good deal of variation between theorists considered here (and some creative ambiguity) with respect to whether they believed the state of nature to be a real state that had ever existed, or merely a rhetorical device to contrast the actual human condition with what we might have been like without formal institutions. Locke took some trouble (at the end of Chapter 2 of the *Second treatise*, for example) to defend the view that it had once really existed. Likewise, Hobbes writes in Chapter 13 of *Leviathan* that ‘during the time men live without a common Power to keep them all in awe, they are in that condition which is called Warre; and such a warre, as is of every man, against every man’; he uses the present tense, not the conditional. In the following section he is then very explicit:

It may peradventure be thought, there was never such a time, nor condition of warre as this; and I believe it was never generally so, over all the world: but there are many places, where they live so now. For the savage people in many places of America, except the government of small Families, the concord whereof dependeth on naturall lust, have no government at all; and live at this day in that brutish manner, as I said before.

Nevertheless, in his arguments Hobbes does not draw on any historical description, but rather on his abstract reflections about the natural equality of men in terms of physical strength and cunning, which leads to ‘diffidence’, by which he means a natural fear of each other. In terms of the way in which modern game-theorists use equilibrium analysis, we can think of Locke as considering the state of nature to be an actual outcome of social arrangements under some real conditions, while Hobbes mainly considered it an ‘out-of-equilibrium’ state, the credible threat of which was enough to persuade rational individuals to grant legitimacy to the sovereign.

Rousseau's view is harder to characterise, since he repeatedly uses ambiguous language. For instance, in Chapter 6 of Book 1 of *The social contract*, he writes ‘I assume that men reach a point where the obstacles to their preservation in a state of nature prove greater than the strength each man has to preserve himself in that state … the only way in which they can preserve themselves is by uniting their separate powers’. He is clearly describing a social process, not an exercise in reflection. On the other hand, he uses the present rather than the past tense and writes ‘I assume’ (‘Je suppose’ in the original French) in a way that suggests at least some tentativeness about whether such a process actually occurred. In a passage from the *Discourse* he even writes ‘Let us begin then by setting facts aside, as they do not affect the question’.

However, Rousseau's later descriptions of the origins of the Roman republic (in Chapter 4 of Book 4) also suggest he believed that his notion of the social contract, even if idealised, captures some real features of how at least some societies had historically been constituted. For all three of our social contract theorists, then, it appears that they hedged their bets about the historical accuracy of the state of nature to which they appealed in their theories, but were inclined to believe that if the evidence were available it would have supported their description.

### 4.2. Was the state of nature conceived as solitary, or merely as lacking in formal institutions?

Once again it is not easy to find an unambiguous answer to this question. Hobbes famously used the term ‘solitary’, and Rousseau describes human nature as ‘entirely complete and solitary’ (‘un tout parfait et solitaire’). Locke does not use the term, and his description of the state of nature does not seem to indicate solitude at all, since men are constantly fighting over resources. Nevertheless, all three theorists seemed to believe that there was a solid foundation to human reason that existed prior to any process of socialisation, and to which appeal could be made to judge the conditions under which human beings were to organise their collective lives.

### 4.3. If the social contract theorists were indeed mistaken about human nature, what difference does it make to their normative conclusions?

From the studies of small-scale societies that we have reviewed, it is clear that many specific assumptions that these thinkers made about life without formal institutions were wrong. More importantly, it may be the whole perspective of the social contract that should be questioned. Indeed, the philosophical basis of social contract theory is to judge political institutions as legitimate only if they pass the (hypothetical) test of being unanimously approved by agents under some specific circumstances (the role of the state of nature being to describe these initial circumstances). In these accounts, unanimous consent has to be given by human beings acting in an independent, rational and to some degree solipsistic way. This intellectual construction, which puts emphasis on formal institutions and on an individualistic, society-free perspective, seems at odds with what we have learned from the evolutionary social sciences including anthropology. First, formal institutions are certainly not the only way to regulate human behaviour: social norms and many other coordinating behaviours exist without formal institutions. Second, everywhere, individuals are interdependent and embedded in a network of complex social relations which shape psychology. Acknowledging this invites us to revisit the notion of contract among solipsistic individuals populating the state of nature, even if it is used as a rhetorical device.

## 5. Conclusions

Taking a broad overview of the accumulated evidence from small-scale societies about the questions that preoccupied the political philosophers whose views we have examined, it is hard not to be struck by the extent to which they underestimated human behavioural complexity in societies that lack formal institutions. Philosophers were not the only thinkers to have such biases: the scientific community as a whole, persuaded of the virtues of codified knowledge and practice in modern social organisation, has often been surprised over many decades by what communities of social organisms (both humans and non-humans) can achieve without formal institutions.

It seems therefore that the deployment of the social contract as a device for philosophical reflection, despite its major value in clarifying what can and should be expected of political institutions, has come at a cost – namely that of underestimating and perhaps undervaluing the resilience and subtlety of human behaviour in societies that do not have the formal institutions on which we have come unthinkingly to rely.

The notion of a state of nature is largely absent from the works of contemporary thinkers of the social contract, perhaps because of the inherent weakness or the poor empirical foundations of this concept. For example, John Rawls (1971) keeps the notion of a form of consent/social contract as the foundation of the legitimacy of political institutions, but the state of nature has been replaced by the much more abstract notion of an ‘original position’. He writes in the first chapter of *A theory of justice* that he wants to propose ‘a theory of justice that generalises and carries to a higher level of abstraction the traditional conception of the social contract. The compact of society is replaced by an initial situation that incorporates certain procedural constraints on arguments designed to lead to an original agreement on principles of justice’. The state of nature has also disappeared from the works of contemporary thinkers who have sought to revisit the concept of social contract as an explicit unanimous agreement among rational individuals. For example, Brian Skyrms (1996) argues for a weaker notion of agreement, as implicitly emerging from the dynamic interactions of the members of society. Here too the notion of ‘state of nature’ has disappeared (even if in Skyrms's arguments, the initial conditions may sometimes play an important role, the interpretation is very different).

Although Charles Darwin did not claim to be an expert on small-scale societies, and did not found an explicit political theory on his empirical convictions about the nature of life in such societies, there is abundant evidence that he was profoundly aware of the intensely social nature of life prior to the agricultural and industrial revolutions, and thought this had been so for long enough to shape human behaviour through natural selection. In one remarkable passage in *The descent of man* he speculates as follows:

When two tribes of primeval man, living in the same country, came into competition, if (other circumstances being equal) the one tribe included a great number of courageous, sympathetic and faithful members, who were always ready to warn each other of danger, to aid and defend each other, this tribe would succeed better and conquer the other. Selfish and contentious people will not cohere and without coherence nothing can be effected. A tribe rich in the above qualities would spread and be victorious over other tribes … thus the social and moral qualities would tend slowly to advance and be diffused throughout the world. (Darwin, 1871, part I, pp. 162–163)

When we reflect on the profound sentence that concludes *The descent of man* (‘Man still bears in his bodily frame the indelible stamp of his lowly origin’ (Darwin, 1871, part II, p. 405), we should be in no doubt that Darwin intended the ‘indelible stamp’ to apply also to human behaviour. However, anyone tempted to read it as a gloomy statement about our animal nature and the way it pollutes our social behaviour, might fruitfully compare it with the closing paragraph of the *The origin of species*, where he writes:

There is grandeur in this view of life, with its several powers, having been originally breathed into a few forms or into one; and that, whilst this planet has gone cycling on according to the fixed law of gravity, from so simple a beginning endless forms most beautiful and most wonderful have been, and are being, evolved. (Darwin, 1859, pp. 459–460).

Modern evolutionary social science has affirmed most emphatically the existence of ‘endless forms most beautiful’ of non-human and human behaviour in small-scale societies, and we believe political philosophy can only be enriched by taking these explicitly into account.

## Research transparency and reproducibility

No data are reported in this paper.

## References

[ref1] Alger, I., Hooper, P., Cox, D., Stieglitz, J., & Kaplan, H. (2020). Paternal provisioning results from ecological change. PNAS, 117, 10746*–*10754. 32358187 10.1073/pnas.1917166117 PMC7245097

[ref2] Alger, I., & Weibull, J. (2019). Evolutionary models of preference formation. Annual Review of Economics, 11, 329*–*354.

[ref3] Ames, K. (2003). The northwest coast. Evolutionary Anthropology, 12, 19*–*33.

[ref4] Barrett, H., Bolyanatz, A., Crittenden, A., Fessler, D., Fitzpatrick, S., Gurven, M., … S.L. (2016). Small-scale societies exhibit fundamental variation in the role of intentions in moral judgment. PNAS, 113, 4688*–*4693. 27035959 10.1073/pnas.1522070113 PMC4855604

[ref5] Betzig, L. (1989). Causes of conjugal dissolution: A cross-cultural study. Current Anthropology, 30, 654*–*676.

[ref6] Bird, D., Bliege Bird, R., Codding, B., & Zeanah, D. (2019). Variability in the organization and size of hunter–gatherer groups: Foragers do not live in small-scale societies. Journal of Human Evolution, 131, 96*–*108. 31182209 10.1016/j.jhevol.2019.03.005

[ref7] Blanchflower, D., & Oswald, A. (2008). Is well-being u-shaped over the life cycle? Social Science and Medicine, 66, 1733*–*1749. 18316146 10.1016/j.socscimed.2008.01.030

[ref8] Boehm, C. (1999). Hierarchy in the forest: The evolution of egalitarian behavior. Harvard University Press.

[ref9] Borgerhoff Mulder, M., Bowles, S., Hertz, T., Bell, A., Beise, J., Clark, G., … Wiessner, P. (2009). Intergenerational wealth transmission and the dynamics of inequality in small-scale societies. Science, 326, 682*–*688. 19900925 10.1126/science.1178336 PMC2792081

[ref10] Boyd, R. (2017). A different kind of animal: How culture transformed our species. Princeton University Press.

[ref11] Boyd, R., & Richerson, P. (1985). Culture and the evolutionary process. University of Chicago Press.

[ref12] Cosmides, L., & Tooby, J. (1992). Cognitive adaptations for social exchange. In J. Barkow, L. Cosmides, & J. Tooby (Eds.), The adapted mind: Evolutionary psychology and the generation of culture (pp. 163–228). Oxford University Press.

[ref13] Darwin, C. (1859). On the origin of species by means of natural selection. John Murray, facsimile edition published in 1979 by Gramercy Books, a division of Random House, New York.

[ref14] Darwin, C. (1871). The descent of man, and selection in relation to sex. John Murray, facsimile edition 1981, Princeton University Press.

[ref15] Derex, M., Bonnefon, J.-F., Boyd, R., & Mesoudi, A. (2019). Causal understanding is not necessary for the improvement of culturally evolving technology. Nature Human Behaviour, 3, 446*–*452. 10.1038/s41562-019-0567-9 30936426

[ref16] Derex, M., & Boyd, R. (2018). Social information can potentiate understanding despite inhibiting cognitive effort. Scientific Reports, 8, 9980. 29967356 10.1038/s41598-018-28306-z PMC6028476

[ref17] de Waal, F. (2017). Peacemaking among primates. Harvard University Press.

[ref18] Durkheim, E. (1915 [2001]). The elementary forms of religious life. Trans. Carol Cosman. Oxford University Press.

[ref19] Engel, C. (2011). Dictator games: A meta study. Experimental Economics, 14, 583*–*610.

[ref20] Fehr, E., & Fischbacher, U. (2004). Third party punishment and social norms. Evolution and Human Behavior, 25(2), 63*–*87, doi:10.1016/S1090-5138(04)00005-4.

[ref21] Fehr, E., & Gächter, S. (2000). Cooperation and punishment in public goods experiments. American Economic Review, 90, 980*–*994.

[ref22] Fehr, E., Gächter, S., & Kirchsteiger, G. (1997). Reciprocity as a contract enforcement device. Econometrica, 65, 833*–*860.

[ref23] Fehr, E., Kirchsteiger, G., & Riedl, A. (1993). Does fairness prevent market clearing? An experimental investigation. Quarterly Journal of Economics, 108, 437*–*460.

[ref24] Fehr, E., & Schmidt, K. (1999). A theory of fairness, competition and co-operation. Quarterly Journal of Economics, 114, 817–868.

[ref25] Fischbacher, U. (2001). Are people conditionally cooperative? Economics Letters, 71(3), 397–404. doi:10.1016/S0165-1765(01)00394-9.

[ref26] Forsythe, R., Horowitz, J., Savin, N., & Sefton, M. (1994). Fairness in simple bargaining games. Games and Economic Behavior, 6, 347*–*369.

[ref27] Fry, D. (2005). The human potential for peace. An anthropological challenge to assumptions about war and violence. Oxford University Press.

[ref28] Garfield, Z., Syme, K., & Hagen, E. (2020). Universal and variable leadership dimensions across human societies. Evolution and Human Behavior, 41(5), 397*–*414.

[ref29] Garfield, Z., von Rueden, C., & Hagen, E. (2019). The evolutionary anthropology of political leadership. The Leadership Quarterly, 30, 59*–*80.

[ref30] Gavrilets, S. (2012). On the evolutionary origins of the egalitarian syndrome. PNAS, 109, 14069*–*14074. 22891349 10.1073/pnas.1201718109 PMC3435232

[ref31] Glowacki, L., Wilson, M. L., & Wrangham, R. W. (2020). The evolutionary anthropology of war. Journal of Economic Behavior and Organization, 178, 963–982.

[ref32] Gurven, M., Blackwell, A., Eid, D., Stieglitz, J., & Kaplan, H. (2012). Does blood pressure inevitably rise with age?: Longitudinal evidence among forager–horticulturalists. Hypertension, 60, 25–33. 22700319 10.1161/HYPERTENSIONAHA.111.189100 PMC3392307

[ref33] Gurven, M., & Kaplan, H. (2007). Longevity among hunter–gatherers: A cross-cultural examination. Population and Development Review, 33, 321–365.

[ref34] Hagen, E., & Tushingham, S. (2019). The prehistory of psychoactive drug use. In T. Henley, M. Rossano, & E. Kardas (Eds.), Handbook of cognitive archaeology (pp. 471–498). Routledge.

[ref35] Henrich, J. (2020). The WEIRDest people in the world: How the West became psychological, peculiar and particularly prosperous. Farrar Strauss and Giroux.

[ref36] Henrich, J., Boyd, R., Bowles, S., Camerer, C., Fehr, E., Gintis, H., & McElreath, R. (2001). In search of Homo economicus: Behavioral experiments in 15 small scale societies. American Economic Review, 91(2), 73–78, doi:10.1257/aer.91.2.73.

[ref37] Henrich, J., & Gil-White, F. J. (2001). The evolution of prestige – Freely conferred deference as a mechanism for enhancing the benefits of cultural transmission. Evolution and Human Behavior, 22, 165–196. 11384884 10.1016/s1090-5138(00)00071-4

[ref38] Henrich, J., McElreath, R., Barr, A., Ensminger, J., Barrett, C., Bolyanatz, A., … Ziker, J. (2006). Science, 312(5781), 1767*–*1770, doi:10.1126/science.1127333. 16794075

[ref39] Hess, N., Helfrecht, C., Hagen, E., Sell, A., & Hewlett, B. (2010). Interpersonal aggression among Aka hunter–gatherers of the Central African Republic. Human Nature, 21, 330–354.

[ref40] Hill, K., Walker, R., Bozicevic, M., Eder, J., Headland, T., Hewlett, B., … Wood, B. (2011). Co-residence patterns in hunter–gatherer societies show unique human social structure. Science, 331, 1286–1289. 21393537 10.1126/science.1199071

[ref41] Hobbes, T. (1651). Leviathan. A. Crooke.

[ref42] Hooper, P., Simon DeDeo, S., Caldwell, A., Gurven, M., & Kaplan, H. (2013). Dynamical structure of a traditional amazonian social network. Entropy, 15, 4932–4955. 25053880 10.3390/e15114932 PMC4104206

[ref43] Jaeggi, A., Blackwell, A., von Rueden, C., Trumble, B., Stieglitz, J., Garcia, A., … Gurven, M. (2020). Relative wealth and inequality associate with health in a small-scale subsistence society. medRxiv preprint, submitted. 10.7554/eLife.59437 PMC8225390 33988506

[ref44] Kaplan, H., Hooper, P., and Gurven, M. (2000). The evolutionary and ecological roots of human social organization. Philosophical Transactions of the Royal Society B, 364, 3289–3299. 10.1098/rstb.2009.0115 PMC2781874 19805435

[ref45] Kaplan, H., Thompson, R., Trumble, B., Wann, S., Allam, A., Beheim, B., … Thomas, G. (2017). Coronary atherosclerosis in indigenous South American Tsimane: A cross-sectional cohort study. The Lancet, 389, 1730–1739. 10.1016/S0140-6736(17)30752-3 PMC6028773 28320601

[ref46] Keynes, J. M. (1936). The general theory of employment, interest and money. Macmillan.

[ref47] Khaldun, I. (1377 [2015]). The Muqaddimah: An introduction to history. Trans. Franz Rosenthal. Princeton University Press.

[ref48] Kirby, K., Godoy, R., Reyes-Garcia, V., Byron, E., Apaza, L., Leonard, W., … Wilkie, D. (2002). Correlates of delay-discount rates: Evidence from Tsimane’ Amerindians of the Bolivian rain forest. Journal of Economic Psychology, 23, 291–316.

[ref49] Kondo, N., Sembajwe, G., Kawachi, I., van Dam, R., Subramanian, S., & Yamagata, Z. (2009). Income inequality, mortality, and self rated health: Meta-analysis of multilevel studies. BMJ, 339, b4471. 19903981 10.1136/bmj.b4471 PMC2776131

[ref50] Kontis, V., Bennett, J., Mathers, C., Li, G., Foreman, K., & Ezzati, M. (2017). Future life expectancy in 35 industrialised countries: projections with a Bayesian model ensemble. The Lancet, 389, 1323–1335. 10.1016/S0140-6736(16)32381-9 PMC5387671 28236464

[ref51] Kuhlwilm, M., Gronau, I., Hubisz, M., de Filippo, C., Prado-Martinez, J., Kircher, M., … Castellano, S. (2016). Ancient gene flow from early modern humans into eastern neanderthals. Nature, 530, 429–433. 26886800 10.1038/nature16544 PMC4933530

[ref52] Kuhn, S., & Stiner, M. C. (2019). Hearth and home in the middle Pleistocene. Journal of Anthropological Research, 75, 305–327.

[ref53] Lee, R. (2014). Intergenerational transfers, social arrangements, life histories, and the elderly. In M. Weinstein, & M. Lane (Eds.), Sociality, hierarchy, health: Comparative biodemography: A collection of papers (pp. 223–246). National Academies Press. 25254285

[ref54] Locke, J. (1689a). Essay concerning human understanding. Thomas Basset.

[ref55] Locke, J. (1689b [1988]). Two treatises of government, Ed. P. Laslett. Cambridge University Press.

[ref56] Macfarlan, S., Remiker, M., & Quinlan, R. (2012). Competitive altruism explains labor exchange variation in a Dominican community. Current Anthropology, 53, 118–124.

[ref57] Macpherson, C. (1962). The political theory of possessive individualism. Clarendon Press.

[ref58] Markus, H., & Kitayama, S. (1991). Culture and the self: Implications for cognition, emotion, and motivation. Psychological Review, 98, 224–253.

[ref59] Marmot, M., Smith, G., Stansfeld, S., Patel, C., North, F., Head, J., … Feeney, A. (1991). Health inequalities among British civil servants: The Whitehall II study. The Lancet, 337, 1387–1393. 10.1016/0140-6736(91)93068-k 1674771

[ref60] Mathew, S. (2017). How the second-order free rider problem is solved in a small-scale society. American Economic Review, 107, 578–581.

[ref61] Mehr, S., Singh, M., Knox, D., Ketter, D., Pickens-Jones, D., Atwood, S., Lucas, C., … Glowacki, L. (2019). Universality and diversity in human song. Science, 366, eaax0868. 31753969 10.1126/science.aax0868 PMC7001657

[ref62] Migliano, A., Page, A., Gómez-Garden˜es, J., Salali, G., Viguier, S., Dyble, M., … Vinicius, L. (2017). Characterization of hunter–gatherer networks and implications for cumulative culture. Nature Human Behaviour, 1, 0043.

[ref63] Morris, I. (2014). War! What is it good for? Conflict and the progress of civilization from primates to robots. Picador.

[ref64] Oeppen, J., & Vaupel, J. (2002). Broken limits to life expectancy. Science, 296, 1029–1031. 12004104 10.1126/science.1069675

[ref65] Peterson, D., & Wrangham, R. (1997). Demonic males: Apes and the origins of human violence. Mariner Books.

[ref66] Pinker, S. (2002). The blank slate. Penguin.

[ref67] Pinker, S. (2011). The better angels of our nature. Viking.

[ref68] Pisor, A., Gervais, M., Purzycki, B., & Ross, C. (2020). Preferences and constraints: the value of economic games for studying human behaviour. Royal Society Open Science, 7, 192090. 32742683 10.1098/rsos.192090 PMC7353969

[ref69] Power, E. (2017a). Discerning devotion: Testing the signaling theory of religion. Evolution and Human Behavior, 38, 82–91.

[ref70] Power, E. (2017b). Social support networks and religiosity in rural South India. Nature Human Behaviour, 1, 0057.

[ref71] Power, E. (2018). Collective ritual and social support networks in rural South India. Proceedings of the Royal Society B, 285, 20180023. 29794040 10.1098/rspb.2018.0023 PMC5998092

[ref72] Purzycki, B., & Arakchaa, T. (2013). Ritual behavior and trust in the Tyva Republic. Current Anthropology, 54, 381–388.

[ref73] Purzycki, B., & Lang, M. (2019). Identity fusion, outgroup relations, and sacrifice: A cross-cultural test. Cognition, 186, 1–6. 30711768 10.1016/j.cognition.2019.01.015

[ref74] Purzycki, B., Pisor, A., Apicella, C., Atkinson, Q., Cohen, E., Henrich, J., … Xygalatas, D. (2018). The cognitive and cultural foundations of moral behavior. Evolution and Human Behavior, 39, 490–501.

[ref75] Rawls, J. (1971). A theory of justice. Harvard University Press.

[ref76] Reyes-Garcia, V., Zurro, D., Caro, J., & Madella, M. (2017). Small-scale societies and environmental transformations: coevolutionary dynamics. Ecology and Society, 22(1), 15.30416527

[ref77] Richerson, P., & Boyd, R. (2005). Not by genes alone: How culture tranformed human evolution. University of Chicago Press.

[ref78] Rousseau, J.-J. (1755 [1984]). A discourse on inequality. Trans. Maurice Cranston. Penguin Classics.

[ref79] Rousseau, J.-J. (1762 [1968]). The social contract. Trans. Maurice Cranston. Penguin Classics.

[ref80] Rowley-Conwy, P. (2001). Time, change and the archaeology of hunter–gatherers: How original is the ‘original affluent society’? In C. Panter-Brick, R. Layton, & P. Rowley-Conwy (Eds.), Hunter–gatherers: An interdisciplinary perspective (pp. 39–72). Cambridge University Press.

[ref81] Sapolsky, R. (2005). The influence of social hierarchy on primate health. Science, 308, 648–652. 15860617 10.1126/science.1106477

[ref82] Saucier, G., Thalmayer, A., & Bel-Bahar, T. (2014). Human attribute concepts: Relative ubiquity across twelve mutually isolated languages. Journal of Personality and Social Psychology, 107, 199–216. 24956320 10.1037/a0036492

[ref83] Scelza, B., Prall, S., Blumenfield, T., Crittenden, A., Gurven, M., Kline, M., … McElreath, R. (2020). Patterns of paternal investment predict cross-cultural variation in jealous response. Nature Human Behaviour, 4, 20–26. 10.1038/s41562-019-0654-y PMC8006540 31332300

[ref84] Schniter, E., Wilcox, N., Beheim, B., Kaplan, H., & Gurven, M. (2018). Information transmission and the oral tradition: Evidence of a late-life service niche for Tsimane Amerindians. Evolution and Human Behavior, 39, 94–105. 34650327 10.1016/j.evolhumbehav.2017.10.006 PMC8513776

[ref85] Shostak, M. (1981). Nisa – The life and words of a !Kung woman. Harvard University Press.

[ref86] Skinner, Q. (1969). Meaning and understanding in the history of ideas. History and Theory, 8, 3–53, doi:10.2307/2504188.

[ref87] Skyrms, B. (1996). Evolution of the social contract. Cambridge University Press.

[ref88] Smith, K., & Apicella, C. (2019). Hadza hunter–gatherers disagree on perceptions of moral character. Social Psychological and Personality Science, 11, 616–625.

[ref89] Smith, K., Larroucau, T., Mabulla, I., & Apicella, C. (2018). Hunter–gatherers maintain assortativity in cooperation despite high levels of residential change and mixing. Current Biology, 28, 3152–3157. 30245106 10.1016/j.cub.2018.07.064

[ref90] Smith, D., Schlaepfer, P., Major, K., Dyble, M., Page, A., Thompson, J., … Migliano, A. (2017). Cooperation and the evolution of hunter–gatherer storytelling. Nature Communications, 8, 1853. 10.1038/s41467-017-02036-8 PMC5717173 29208949

[ref91] Snyder-Mackler, N., Burger, J., Gaydosh, L., Belsky, D., Noppert, G., Campos, F., … Tung, J. (2020). Social determinants of health and survival in humans and other animals. Science, 368, eaax9553. 32439765 10.1126/science.aax9553 PMC7398600

[ref92] Stieglitz, J., Gurven, M., Kaplan, H., & Hopfensitz, A. (2017). Why household inefficiency? An experimental approach to assess spousal resource distribution preferences in a subsistence population undergoing socioeconomic change. Evolution and Human Behavior, 38, 71–81.

[ref93] Stieglitz, J., Jaeggi, A., Blackwell, A., Trumble, B., Gurven, M., & Kaplan, H. (2014). Work to live and live to work: Productivity, transfers, and psychological well-being in adulthood and old age. In M. Weinstein, & M. Lane (Eds.), Sociality, hierarchy, health: Comparative biodemography: A collection of papers (pp. 197–221). National Academies Press. 25254285

[ref94] Stieglitz, J., Schniter, E., von Rueden, C., Kaplan, H., & Gurven, M. (2015). Functional disability and social conflict increase risk of depression in older adulthood among bolivian forager–farmers. Journals of Gerontology: Social Sciences, 70, 948–956. 10.1093/geronb/gbu080 PMC4841159 24986182

[ref95] Sugiyama, L., Tooby, J., and Cosmides, L. (2002). Cross-cultural evidence of cognitive adaptations for social exchange among the shiwiar of ecuadorian amazonia. PNAS, 99, 11537–11542. 12177409 10.1073/pnas.122352999 PMC123291

[ref96] Sznycer, D., Xygalatas, D., Agey, E., Alami, S., An, X., Ananyeva, K., … Tooby, J. (2018). Cross-cultural invariances in the architecture of shame. PNAS, 115, 9702–9707. 30201711 10.1073/pnas.1805016115 PMC6166838

[ref97] Tacey, I., & Riboli, D. (2014). Violence, fear and anti-violence: the Batek of peninsular Malaysia. Journal of Aggression, Conflict and Peace Research, 6, 203–215.

[ref98] Trumble, B., Cummings, D., von Rueden, C., O'Connor, K., Smith, E., Gurven, M., & Kaplan, H. (2012). Physical competition increases testosterone among amazonian forager–horticulturalists: A test of the ‘challenge hypothesis’. Proceedings of the Royal Society B, 279, 2907–2912. 22456888 10.1098/rspb.2012.0455 PMC3367794

[ref99] Tully, J. (1980). TA discourse on property: John Locke and his adversaries. Cambridge University Press.

[ref100] Tully, J. (1993). An approach to political philosophy: Locke in contexts. Cambridge University Press.

[ref101] Wiessner, P. (1982). Risk, reciprocity and social influences on !kung san economics. In E. Leacock, & R. Lee (Eds.), Politics and history in band societies (pp. 61–84). Cambridge University Press.

[ref102] Wiessner, P. (2005). Norm enforcement among the Ju/’Hoansi bushmen. Human Nature, 16, 115–145. 26189619 10.1007/s12110-005-1000-9

[ref103] Wiessner, P. (2014). Embers of society: Firelight talk among the Ju/’Hoansi bushmen. PNAS, 111, 14027–14035. 25246574 10.1073/pnas.1404212111 PMC4191796

[ref104] Winking, J., & Mizer, N. (2013). Natural-field dictator game shows no altruistic giving. Evolution and Human Behavior, 34, 288–293.

[ref105] Wrangham, R. (2019). The goodness paradox: The strange relationship between virtue and violence in human evolution. Vintage Books.

[ref106] Xygalatas, D., Mitkidis, P., Fischer, R., Reddish, P., Skewes, J., Geertz, A., … Bulbulia, J. (2013). Extreme rituals promote prosociality. Psychological Science, 24, 1602–1605. 23740550 10.1177/0956797612472910

